# ZDHHC5 interacts physically and functionally with DLG1 at primary cilia and regulates ciliary length and kidney morphology

**DOI:** 10.3389/fcell.2026.1805468

**Published:** 2026-06-10

**Authors:** Csenge Kata Rezi, Canan Doganli, Mariam G. Aslanyan, Mohamed Chamlali, Benjamin Mary, Nina Lykke Callesen, Rokeah Rafat Kamel Abu-Khesha, Gaurav D. Diwan, Robert B. Russell, Esben Lorentzen, Karsten Boldt, Ronald Roepman, Lars Allan Larsen, Lotte B. Pedersen

**Affiliations:** 1 Department of Biology, University of Copenhagen, Copenhagen, Denmark; 2 Department of Cellular and Molecular Medicine, University of Copenhagen, Copenhagen, Denmark; 3 Department of Human Genetics, Research Institute for Medical Innovation, Radboud University Medical Center, Nijmegen, Netherlands; 4 BioQuant, Heidelberg University, Heidelberg, Germany; 5 Biochemistry Center (BZH), Heidelberg University, Heidelberg, Germany; 6 Department of Molecular Biology and Genetics - Protein Science, Aarhus University, Aarhus, Denmark; 7 Institute for Ophthalmic Research and Core Facility for Medical Proteomics, Eberhard Karl University of Tübingen, Tübingen, Germany; 8 College of Health Sciences, VinUniversity, Hanoi, Vietnam

**Keywords:** DLG1, glomeruli, primary cilia, proximity-labeling proteomics, SCAMP3, STXBP1, ZDHHC5, zebrafish

## Abstract

**Introduction:**

Primary cilia are critical sensory organelles whose structure and composition are tightly regulated. The Scribble polarity complex protein DLG1 has recently been implicated in controlling both ciliary protein composition and length in mouse kidney epithelial cells. In addition, the palmitoyl transferase ZDHHC5 localizes to primary cilia and negatively regulates their length. However, the molecular mechanisms underlying DLG1-mediated ciliary regulation and its interaction network remain poorly defined.

**Methods:**

To identify cilium-associated DLG1 interactors, mouse inner medullary collecting duct (IMCD3) cells stably expressing DLG1-BioID2 were generated and subjected to proximity-dependent biotinylation proteomics in ciliated conditions. Candidate interactions were validated using immunoprecipitation, AlphaFold structural modeling, and quantitative immunofluorescence microscopy. Functional relevance was further assessed in vivo using zebrafish models with depletion of zdhhc5 paralogs.

**Results:**

Proximity labeling identified 46 high-confidence DLG1 interactors, of which 35 were previously associated with ciliary or centrosomal protein networks. Among these, ZDHHC5 was confirmed as a DLG1 interactor by immunoprecipitation, and structural modeling supported a potential direct interaction. Quantitative imaging demonstrated that ZDHHC5 localizes to primary cilia independently of DLG1. Additional analyses revealed that DLG1 also interacts with SCAMP3 and STXBP1 and is required for proper localization of STXBP1 at the ciliary base. In zebrafish, depletion of zdhhc5a and zdhhc5b resulted in ciliopathy-like phenotypes, including brain edema, curved body axis, and enlarged glomerular structures, accompanied by significantly elongated cilia in both the brain and pronephros.

**Discussion:**

These findings expand the DLG1 interactome and identify key cilia-associated partners, including ZDHHC5 and STXBP1, that may contribute to the regulation of ciliary structure and function. The data support a model in which DLG1 modulates ciliary length and signaling through specific protein–protein interactions, with disruption of this network leading to ciliopathy-related phenotypes. Together, this study provides new mechanistic insight into polarity complex-mediated control of primary cilia in kidney epithelial cells.

## 1 Introduction

Primary cilia are antenna-like sensory organelles present on the surface of many vertebrate cell types, including kidney epithelial cells, where they regulate key signaling pathways such as Sonic hedgehog and G-protein coupled receptor signaling, as well as signaling mediated by the Polycystin-1 and Polycystin-2 complex (Anvarian et al., 2019; Wachten and Christensen, 2025). Receptors, ion channels and downstream effectors of these pathways localize dynamically to the ciliary compartment, and failure to appropriately regulate their ciliary localization is associated with dysregulated signaling. This, in turn, causes diseases known as ciliopathies, which amongst other organs affect the brain and kidneys (Reiter and Leroux, 2017; Mill et al., 2023; Devlin et al., 2025). Prominent ciliopathies affecting the kidney include autosomal dominant (AD) and autosomal recessive (AR) polycystic kidney disease, caused by pathogenic variants in the genes encoding Polycystin-1/2 and Fibrocystin respectively, as well as nephronophthisis (NPH) and Bardet-Biedl syndrome (BBS) (Devlin et al., 2025). In addition, accumulating evidence links mutations in ciliary genes to congenital anomalies of the kidney and urinary tract (CAKUT) (Gabriel et al., 2018), underscoring the importance of primary cilia for kidney development and function.

Primary cilia consist of a membrane-enclosed, microtubule-based axoneme extending from the basal body, which anchors the cilium to the plasma membrane *via* transition fibers (Breslow and Holland, 2019). These fibers, also known as distal appendages, play a key role in ciliary assembly and maintenance, as they serve as docking sites for the intraflagellar transport (IFT) machinery (Deane et al., 2001) - a bidirectional, microtubule-based transport system essential for the assembly and maintenance of nearly all cilia types (Kozminski et al., 1993; Lacey and Pigino, 2025) - as well as for cytoplasmic vesicles carrying ciliary membrane and axonemal components (Wood and Rosenbaum, 2014; Hu and Harris, 2020; Zhao et al., 2023; Kanie et al., 2025). The transition fibers also cooperate with the transition zone, located between the basal body and cilium proper, to gate ciliary protein and lipid access and thereby help establish and maintain the cilium’s unique protein and lipid composition (Nachury and Mick, 2019; Park and Leroux, 2022; Moran et al., 2024). Regulation of ciliary composition further relies on the IFT machinery and its associated cargo adapter complexes such as Tubby-like proteins and the BBSome - an octameric complex of BBS-associated proteins that facilitates the retrieval of ubiquitinated membrane proteins from cilia by coupling them to the IFT machinery during retrograde transport (Nachury et al., 2007; Loktev et al., 2008; Lechtreck et al., 2009; Eguether et al., 2014; Wingfield et al., 2018; Shinde et al., 2023). The retrograde IFT machinery can independently promote retrieval of ubiquitinated proteins from cilia *via* the adapter protein and ubiquitin reader CFAP36 (Lange et al., 2025), while the ESCRT-0 protein HGS was implicated in downregulation of ciliary Polycystin-2 levels in *C. elegans* male sensory neurons (Hu et al., 2007). HGS also accumulated within primary cilia of *Ift27/Bbs19* mutant mouse inner medullary collecting duct (IMCD3) cells or mouse cortical collecting duct (mCCD) cells devoid of the ciliary kinesin-3 motor protein KIF13B (Rezi et al., 2025). Interestingly, mCCD cells lacking KIF13B also displayed time-dependent alterations in ciliary Polycystin-2 levels, suggesting that KIF13B functions downstream of the BBSome to mediate endocytic retrieval of Polycystin-2 and other ciliary components (Rezi et al., 2025).

While several mechanisms are involved in mediating retrieval and downregulation of ciliary membrane proteins such as Polycystin-2, their delivery to the cilium is also subject to complex regulatory pathways. For example, current evidence indicates that in polarized kidney epithelial cells post-Golgi vesicles carrying Polycystin-2 are first delivered to the plasma membrane or recycling endosomes before being transported to the ciliary base. There, these vesicles dock at transition fibers prior to entry of Polycystin-2 into the cilium (Monis et al., 2017; Walker et al., 2019; Hu and Harris, 2020). Supporting the role of recycling endosomes in ciliary Polycystin-2 trafficking, disruption of proteins associated with these compartments - such as retromer components, the BLOC-1 complex, or Rab GTPases RAB8 and RAB11 - significantly reduces Polycystin-2 levels in cilia (Hoffmeister et al., 2011; Yu et al., 2016; Monis et al., 2017; Tilley et al., 2018). Similarly, we recently showed that kidney epithelial cells lacking Discs large MAGUK scaffold protein 1 (DLG1) display reduced ciliary levels of Polycystin-2, likely because DLG1 functions upstream of the retromer-associated protein SDCCAG3 and IFT20 to promote vesicular trafficking to cilia (Rezi et al., 2024). However, the exact molecular mechanisms by which DLG1 regulates protein trafficking to cilia are not fully understood.

DLG1 is a multifunctional scaffold protein belonging to the membrane-associated guanylate kinase homolog (MAGUK) family, characterized by the ability to organize signaling complexes at specific membrane domains. Structurally, DLG1 contains an N-terminal LIN-2,-7 (L27) domain, three postsynaptic density-95/discs large/zona occludens-1 (PDZ) domains, a SRC homology 3 (SH3) domain, and a C-terminal guanylate kinase (GUK)-like domain that is catalytically inactive. This domain composition enables DLG1 to serve as a versatile scaffold, mediating interactions with a wide range of proteins and thereby influencing diverse cellular processes (Ye et al., 2018). DLG1 was originally identified in *Drosophila melanogaster*, where it plays a critical role in establishing and maintaining apical-basal polarity in epithelial tissues. In these cells, DLG1 localizes to the basolateral membrane beneath the adherens junctions and forms a polarity complex with SCRIB and LGL, which is essential for epithelial integrity and tissue organization. This function is evolutionarily conserved, highlighting the importance of DLG1 in maintaining cellular architecture across species (Bilder et al., 2003; Campanale et al., 2017).

Beyond its role in epithelial polarity, DLG1 is involved in numerous signaling pathways and structural processes, reflecting its extensive interaction network. For example, in neurons, DLG1 is found at both presynaptic and postsynaptic sites, where it orchestrates the localization and clustering of ionotropic glutamate receptors and potassium channels thereby serving as a key regulator of neuronal connectivity and excitability. These effects are mediated through its ability to link membrane receptors with intracellular signaling proteins, thereby modulating synaptic strength and plasticity (Tiffany et al., 2000; Sans et al., 2001; Mauceri et al., 2007; Gardoni et al., 2007; Lickert and Van Campenhout, 2012).

Emerging evidence suggests that DLG1 may also associate with and function at primary cilia and centrosomes. For instance, in HT1299 cells, DLG1 was reported to localize to mitotic centrosomes in a PTEN-NEK6-Eg5-dependent manner (van Ree et al., 2016) while proteomic analyses identified DLG1 within the ciliary compartment of IMCD3 cells (Mick et al., 2015; Kohli et al., 2017) and in photoreceptor outer segments, which represent specialized primary cilia (Datta et al., 2015). Consistent with a ciliary role for DLG1, we recently reported that knockout (KO) of *Dlg1* in IMCD3 or mCCD cells leads to altered ciliary protein composition, including reduced levels of SDCCAG3, IFT20, as well as Polycystin-2. Additionally, loss of DLG1 was accompanied by significant ciliary elongation in polarized (filter-grown) mCCD cells but not IMCD3 cells, suggesting context-dependent regulation of ciliary length by DLG1. Consistently, the increased ciliary length phenotype as well as reduced ciliary content of IFT20 and SDCCAG3 were also observed in a kidney-specific *Dlg1* KO mouse model (Rezi et al., 2024).

To further explore the role of DLG1 in regulating ciliary protein content, we characterized its interactome in ciliated IMCD3 cells using an unbiased, DLG1-BioID2-mediated proximity labeling approach. We identified 46 putative, high-confidence DLG1 interactors, including both well-established DLG1 partners and several proteins previously associated with cilia or centrosomes. Notably, this latter group includes the palmitoyl transferase ZDHHC5 and STXBP1, which we show localize to primary cilia with DLG1 being required for ciliary presence of the latter. Moreover, using zebrafish as an *in vivo* model, we provide evidence that ZDHHC5 contributes to ciliary length regulation and development of the brain and kidney.

## 2 Materials and methods

### 2.1 Cloning

The DLG1-BioID2 plasmid was generated by cloning the rat *Dlg1* coding sequence (Wu et al., 1998) using standard Gateway cloning techniques. As per the manufacturer’s instructions, the *Dlg1* coding sequence was initially inserted into pDONR201 *via* a BP reaction, and the resulting entry vector was then subcloned into a modified pgLAP5 destination vector containing a BioID2 sequence for C-terminal tagging through an LR reaction.

### 2.2 Cell lines and culturing

For proximity labeling experiments and mass spectrometry, the wild type/parental IMCD3 Flp-In cells were provided by Maxence Nachury from the University of California, San Francisco (UCSF), United States of America (Liew et al., 2014), and were stably transfected with Thermo Fisher Scientific’s Flp-In™ technology to express DLG1-BioID2, as detailed in (Aslanyan et al., 2023). As a control, IMCD3 Flp-In cells expressing only BioID2 were used; these cells were previously characterized in (Aslanyan et al., 2023). To validate the protein candidates identified from the mass spectrometry dataset, we used the IMCD3 Flp-In cells stably expressing cilia-BioID2 wild type, *Dlg1* ^−/−^, and *Dlg1* ^−/−^ with mCherry-DLG1 rescue lines, as described previously in (Aslanyan et al., 2023; Rezi et al., 2024). IMCD3 cells were cultured in DMEM/F-12, GlutaMAX Supplement (Gibco, cat #31331-093) medium containing 10% fetal bovine serum (FBS; Gibco, cat #10438-026), and 1% Penicillin-Streptomycin (Sigma-Aldrich, cat #P0781), and were maintained at 37 °C in a humidified atmosphere with 5% CO_2_. To induce ciliogenesis, cells were grown to approximately 80% confluence and subsequently cultured for 24 h in DMEM/F-12 without supplements.

HEK293T cells were originally purchased from the American Type Culture Collection (ATCC) (cat #CRL-3216) and were grown in a 37 °C incubator with 5% humidified CO_2_ in high-glucose DMEM (Gibco, cat #41966-052) with 10% FBS (Gibco, cat #10438-026) plus 1% Penicillin-Streptomycin (Sigma-Aldrich, cat #P0781).

### 2.3 Proximity-labeling proteomics

The proximity labeling experiments using the IMCD3 DLG1-BioID2 and BioID (negative control) cell lines were performed in parallel with those described in (Rezi et al., 2024) and hence used the same negative control cell line. Cells were seeded in 15 cm dishes and maintained in standard culture medium consisting of DMEM/F-12 with GlutaMAX Supplement (Gibco, cat #31331-093), supplemented as previously outlined. When cultures reached approximately 80% confluence, ciliogenesis was induced by incubating the cultures for an additional 48 h in DMEM/F-12 – GlutaMaX medium without supplements. Proximity labeling was initiated overnight by adding 10 μM biotin (Sigma-Aldrich, cat #B4501) to the medium. Following labeling, cells were lysed, and samples were processed for mass spectrometry following the BioID2-based protocol published by (Aslanyan et al., 2023; Rezi et al., 2024).

Protein identification was carried out following the protocol described by (Aslanyan et al., 2023). For downstream proteomics analysis, we employed a custom R script developed in-house, which mirrors the functionality of Perseus software (Tyanova et al., 2016a). Label-free quantification (LFQ) intensity values were compared between DLG1-BioID2 and BioID2 samples. For replicates where LFQ intensity was zero in fewer than half of the samples but present in others, missing values were imputed from a normal distribution with a mean 1.8-fold lower than the mean of observed values and a standard deviation equal to 0.5 times that mean. Statistical significance was assessed using Student’s t-test with p-value correction using the Benjamini–Hochberg method and the Significance A test to detect proteins with extreme log_2_ ratios. Proteins significantly altered in the DLG1-BioID2 *versus* the BioID2 control comparison were categorized into two tiers:

Tier 1: Proteins with Benjamini–Hochberg corrected *t*-test p-values (i.e., q-values) < 0.05 and Significance A p-values <0.05.

Tier 2: Proteins with only corrected *t*-test p-values (q-values) < 0.05.

### 2.4 Immunofluorescence microscopy of cell lines

IMCD3 cells were plated and cultured on 12-mm glass coverslips. When cells reached approximately 80% confluence, they were serum-starved for 24 h in the previously described medium to promote ciliogenesis. Coverslips were pre-fixed in 0.4% paraformaldehyde (PFA; VWR, cat #9713) for 5 min at 37 °C, followed by permeabilization with PHEM buffer supplemented with 0.5% Triton-X100 for 2 min at 37 °C. After permeabilization, cells were fixed with 4% PFA for 12 min at room temperature, then washed with PBS. The fixed cells were blocked with 3% (w/v) BSA solution for an hour, then incubated with primary antibodies diluted in 3% BSA for 1.5–2 h at room temperature or overnight at 4 °C. Following thorough PBS washes, secondary antibodies diluted in 3% BSA were applied for 1 h at room temperature. Nuclei were counterstained with DAPI (Sigma-Aldrich, cat #D9542). Details of antibodies and dilutions used for immunofluorescence microscopy (IFM) are provided in Table 1. Coverslips were mounted using a homemade mounting medium consisting of 6% N-propyl gallate (Sigma-Aldrich, cat #P3130) mixed with ShandonTM Immu-Mount (Epredia, cat #9990402) at a 1:12 ratio.

**TABLE 1:** Reagents and tools used in this study.

Reagent/Resource	Reference or source	Identifier or catalog number
Experimental models		
IMCD3 Flp-In cell line (*M. musculus*)	Maxence Nachury; (Liew et al., 2014)	Wild type (parental)
IMCD3 Flp-In w/BioID2 cell line (*M. musculus*)	Aslanyan et al. (2023)	Wild type with BioID2 at Flp-In locus
IMCD3 Flp-In w/DLG1-BioID2 cell line (*M. musculus*)	This study	Wild type with DLG1-BioID2 at Flp-In locus
HEK293T cell line (*H. sapiens*)	ATCC	cat #CRL-3216
IMCD3 Flp-In w/cilia-BioID2 cell line (*M. musculus*)	Aslanyan et al. (2023)	Wild type with NPHP3 (aa 1-203)-BioID2 at Flp-In locus
IMCD3 Flp-In w/cilia-BioID2 *Dlg1* ^*−/−*^ cells (*M. musculus*)	Rezi et al. (2024)	Pool of knockout cells
IMCD3 Flp-In w/cilia-BioID2 *Dlg1* ^*−/−*^ w/mCherry-DLG1 cells (*M. musculus*)	Rezi et al. (2024)	Pool/rescue line
DH10B cell line (*E. coli*)	Lab stock	N/A
Recombinant DNA		
pEGFP-C1	Clontech	GFP
*Rattus norvegicus* DLG1 in pEGFP-C1	Wu et al. (1998)	GFP-DLG1
*Homo sapiens* KIF13B residues 561-1826 (Tail 6) in pEGFP-C1	Schou et al. (2017)	GFP-KIF13B Tail 6
pgLAP5-BioID2-N1	Aslanyan et al. (2023)	Flp-In expression plasmid
*Rattus norvegicus* DLG1 in pDONR201 w/o STOP codon (for C-term tagging)	This study	pDONR201-DLG1
*Rattus norvegicus* DLG1 in pgLAP5-BioID2 (C-tagged)	This study	DLG1-BioID2
pOG44	Hallacli et al. (2022)	Flp-Recombinase Expression Vector
*M. musculus* ZDHHC5 in pNeo-Flag	Addgene; (Tian et al., 2015)	cat #85812
*H. sapiens* UNC119B in pCIneoMyc	Addgene; (Iwasa et al., 2018)	cat #128333
Antibodies and dilutions		
Anti-alpha-tubulin (mouse monoclonal); WB (1:10,000)	Sigma-Aldrich	cat #T5168
Anti-acetylated α-tubulin (mouse monoclonal); IFM (1:2,000)	Sigma-Aldrich	cat #T7451
Anti-acetylated α-tubulin (rabbit monoclonal); IFM (1:2,000)	Abcam	cat #ab179484
Anti-BioID2 (chicken); WB (1:5000); IFM (1:2,000)	BioFront Technologies	BID2-CP-100
Anti-DLG1 (rabbit polyclonal); WB (1:1,000)	Abcam	cat #ab300481
Anti-FLAG (mouse monoclonal); WB (1:1,000)	Sigma-Aldrich	cat #F1804
Anti-GAPDH (rabbit polyclonal); WB (1:1,000)	Cell Signaling Technology	cat #2118
Anti-GFP (rabbit polyclonal); WB (1:500)	Sigma-Aldrich	cat #SAB4301138
Anti-GFP (chicken polyclonal); IFM (1:500)	Abcam	cat #ab13970
Anti-GFP (rabbit polyclonal); IFM (1:500)	Novus Biologicals	cat #NB600-308
Anti-MYC (mouse monoclonal); WB (1:1,000)	Cell Signaling	cat #2276
Anti-Polyglutamylation Modification (mouse monoclonal); IFM (1:500)	AdipoGen Life Sciences	cat #AG-20B-0020-C100
Anti-SCAMP3 (rabbit polyclonal); WB (1:500)	Sigma-Aldrich	cat #HPA071167
Anti-STXBP1 (rabbit polyclonal); WB (1:500); IFM (1:500)	Proteintech	cat #11459-1-AP
Anti-ZDHHC5 (rabbit polyclonal); WB (1:500); IFM (1:500)	Proteintech	cat #21324-1-AP
Anti-Chicken-AF488 (donkey polyclonal); WB (1:500)	Invitrogen	cat #A-11039
Anti-Mouse-AF568 (donkey polyclonal); IFM (1:600)	Invitrogen	cat #A-10037
Anti-Rabbit-AF488 (donkey polyclonal); IFM (1:600)	Invitrogen	cat #A-21206
Goat anti-rabbit IgG (H + L) Alexa Fluor™ 488; IFM (1:500)	Invitrogen	cat #A-11008
Goat anti-mouse IgG (H + L) Alexa Fluor™ 594; IFM (1:500)	Invitrogen	cat #A-11032
Anti-Mouse-HRP (goat polyclonal); WB (1:10,000)	Agilent Technologies, Inc.	cat #P0447
Anti-Rabbit-HRP (swine polyclonal); WB (1:10,000)	Agilent Technologies, Inc.	cat #P0399
Alexa Fluor 488 Goat Anti-Chicken; IFM (1:500)	Jackson ImmunoResearch	cat #103-545-155
Alexa Fluor 594 Goat Anti-Mouse; IFM (1:500)	Jackson ImmunoResearch	cat #115-585-003
Chemicals, enzymes and other reagents		
Biotin	Sigma-Aldrich	cat #B4501
DAPI; IFM (1:5000)	Sigma Aldrich	cat #D9542
DMEM, high glucose, pyruvate	Gibco	cat #41966029
DMEM/F-12, GlutaMAX Supplement	Gibco	cat #31331-093
Epredia Immu-Mount	Epredia	cat #9990402
Fetal Bovine Serum (FBS)	Gibco	cat #10438-026
Gateway BP Clonase II Enzyme mix	Invitrogen	cat #11789020
Gateway LR Clonase II Enzyme mix	Invitrogen	cat #11791020
Hygromycin B	Gibco	cat #10687010
Lipofectamine 3000 Transfection Reagent	Invitrogen	cat #L3000015
N-propyl-gallate	Sigma-Aldrich	cat #P3130
Paraformaldehyde, 4%	VWR	cat #9713
Penicillin-Streptomycin	Sigma-Aldrich	cat #P0781
Streptavidin, Alexa Fluor 488 Conjugate; IFM (1:1,000)	Invitrogen	cat #S32354
Software		
Adobe Illustrator 2023	Adobe	​
Adobe Photoshop 2023	Adobe	​
AlphaFold v2.1.0	Jumper et al., 2021; Evans et al., 2022	​
cellSens 1.18	Olympus Life Science	​
Elements AR 5.21	Nikon	​
Fiji	Schindelin et al. (2012)	​
GraphPad Prism 10.0.1	GraphPad	​
Perseus	Tyanova et al. (2016b)	​
PyMOL v2.5	Schrodinger LLC, https://pymol.org	​
topGO package in R	Alexa and Rahnenfuhrer, (2023)	​

Images were captured using either an Olympus BX63 upright microscope equipped with a DP72 color camera (12.8 megapixels, 4,140 × 3,096 resolution) and an Olympus UPlanSApo 60× oil immersion objective. Alternatively, we used an Olympus IX83 inverted microscope fitted with a Yokogawa CSU-W1 confocal scanner, a Photometrics Prime 95B CMOS camera, and an Olympus UPlanSApo 60× oil immersion objective. Image processing for publication involved constrained iterative deconvolution using cellSens 1.18 software, followed by montage assembly in Fiji and Adobe Photoshop 2023. Quantitative analysis of ciliary staining intensities was done as described previously (Rezi et al., 2024).

### 2.5 Immunoprecipitation and western blot analysis

Transient transfection and immunoprecipitation (IP) analysis in HEK293T cells were performed following the protocol described by (Goncalves et al., 2021), with the modification that the wash buffer contained 0.1% NP-40 instead of 0.5%. Input and immunoprecipitated fractions were analyzed by SDS-PAGE and western blotting as outlined in (Goncalves et al., 2021), using the antibodies and dilutions listed in Table 1. SDS-PAGE and Western blotting of IMCD3 cell lysates were performed similarly.

### 2.6 Structural modeling

AlphaFold2-Multimer v3 predictions were performed using the full-length sequences of mouse DLG1 (UniProt Q811D0) and mouse ZDHHC5 (UniProt Q8VDZ4). Structural confidence was assessed using ipSAE calculated from PAE values with a 10Å cutoff (Dunbrack, 2025), per-residue pLDDT scores, and interface PAE analysis. The ipSAE metric is designed to be robust to intrinsically disordered regions by filtering out high-PAE residues (>10Å) and focusing on genuine interface contacts (Dunbrack, 2025). We also analyzed per-residue interface metrics to complement the global ipSAE score.

Interface residues were defined using dual criteria: (i) PAE < 4Å between residue pairs, and (ii) inter-residue atomic distance ≤8Å in the 3D model, calculated as the minimum distance between any pair of atoms from the two residues. This combined approach ensures that only residues with both high predicted accuracy and genuine spatial proximity are classified as interface residues. Interface pLDDT was calculated as the mean pLDDT of all residues meeting both criteria. Structure visualization and interface analysis were performed using custom Python scripts and structural figures done using PyMOL.

### 2.7 Zebrafish husbandry and generation of F0 crispants

Zebrafish *Tg(wt1b:GFP)* lines were obtained from the EZRC. Embryos were maintained and staged as previously described (Kimmel et al., 1995) and all experiments were approved and conducted according to licenses and guidelines from the Danish Animal Experiments Inspectorate.

To generate biallelic F0 KOs, three crRNAs were designed for *zdhhc5a* (ENSDART00000003497.11), targeting exons 1, 4 and 7, and additional crRNAs were designed for *zdhhc5b* (ENSDART00000128508.3), targeting exons 1, 4 and 5. Three scrambled crRNAs were used as a control. Cas9/guide RNA (gRNA) ribonucleoprotein (RNP) complexes were assembled following (Kroll et al., 2021) and 14.3 μM RNPs were used for 1 nL injections. All CRISPR–Cas9 components were purchased from Integrated DNA Technologies (IDT). Efficient genome editing was verified by PCR spanning the targeted regions using genomic DNA extracted from injected larvae and relevant primers (Supplementary Figure S5; Supplementary Table S1).

Embryos from *Tg(wt1b:GFP)* breedings were injected with either the three RNPs targeting *zdhhc5a*, the three RNPs targeting *zdhhc5b*, or a combination of both sets; in parallel, control RNPs were injected as negative controls. Gross morphological phenotypes were assessed using a Zeiss Stemi 305 stereomicroscope. Data were presented using GraphPad Prism software (version 9).

### 2.8 Quantification of glomeruli size in F0 crispants

RNPs injected *Tg(wt1b:GFP)* zebrafish embryos were anesthetized in 0.016% tricaine and embryos were imaged dorsally on 3% methyl cellulose by Zeiss AxioZoom V16. Glomerular area measurements were done using Fiji software and plotted using GraphPad Prism software.

### 2.9 Quantification of cilia length in the zebrafish pronephric duct and brain

To analyze cilia in the pronephric duct, RNPs injected *Tg(wt1b:GFP)* zebrafish embryos were fixed in 4% paraformaldehyde (PFA) overnight at 4 °C, followed by stepwise dehydration into methanol for storage in −20 °C. Embryos were gradually rehydrated by washing in PBST (PBS +0.1% Tween-20) four times for 5 min each. After blocking for 2 h in PBS containing 1% DMSO and 10% sheep serum, embryos were incubated overnight at 4 °C with anti-Polyglutamylation Modification, mAb (GT335) (1:500; AdipoGen Life Sciences, AG-20B-0020-C100) and anti-GFP (1:500; Abcam, cat #ab13970). They were then incubated overnight at 4 °C with secondary antibodies Alexa Fluor 594 Goat Anti-Mouse (1:500; Jackson ImmunoResearch, cat #115-585-003) and Alexa Fluor 488 Goat Anti-Chicken (1:500; Jackson ImmunoResearch, cat #103-545-155). Following antibody incubations, embryos were washed and DAPI staining solution (1 μg/mL) was added for 45 min. Embryos were washed with PBST and yolk was removed. Embryos were mounted ventral side down on slides in Vectashield Antifade DAPI Mounting Media. Zeiss LSM 980 confocal microscope was used for imaging (63× objective).

A similar protocol was used for analysis of cilia length in the brain. In this case, RNPs-injected *Tg(arl13b:EGFP)* zebrafish embryos were analyzed. Following rehydration, embryos were permeabilized in 1% Triton X-100 for 1 h and subsequently blocked in 5% goat serum, 0.3% Triton X-100, and 1% DMSO for 4 h at room temperature. Embryos were then incubated overnight at 4 °C with primary antibodies against GFP (1:500; Novus Biologicals, cat #NB600-308) and acetylated α-tubulin (1:200; Sigma-Aldrich, cat #T7451). After washing in wash buffer (1.5% goat serum, 0.2% Triton X-100, 1% DMSO), embryos were incubated with secondary antibodies goat anti-rabbit IgG (H + L) Alexa Fluor 488 (1:500; Invitrogen, cat #A-11008) and goat anti-mouse IgG (H + L) Alexa Fluor 594 (1:500; Invitrogen, cat #A-11032). Following additional washes in 0.3% Triton X-100, embryos were counterstained with DAPI (1 μg/mL) for 1 h, washed in PBST, and mounted laterally on glass-bottom dishes. Imaging was performed using an Olympus IX83 inverted microscope, equipped with a Yokogawa CSU-W1 confocal scanner unit, ORCA-Flash4.0 V3 Digital CMOS camera (type number: C13440-20CU), and Olympus UPlanSApo 100× oil microscope objective.

For the pronephric duct, quantitative analysis of ciliary length was done using the CiliaQ plugin. Briefly, for each image, maximum intensity projections of small stacks were generated. Using CiliaQ Preparator v0.1.2 plugin, masks of cilia were generated using the following parameters: “Segmentation style: Set intensities above thresholds to maximum possible intensity value (creates a binary image) – Channel duplicated to include a copy of the channel that is not segmented. Subtract Background: 10. Additional blur with Gaussian:0.500 - Segmentation method: applying intensity threshold based on the Moments threshold algorithm”. Background and unwanted detected objects were removed with the CiliaQ Editor v0.0.3 plugin. Cilia length measurements were obtained using CiliaQ v0.1.7. For the brain, maximum projection of z stacks were generated, cilia were manually selected using the magic wand tool in ImageJ and their area was measured by the software.

## 3 Results

### 3.1 DLG1 interacts physically with ZDHHC5, STXBP1 and SCAMP3

**FIGURE 1:**
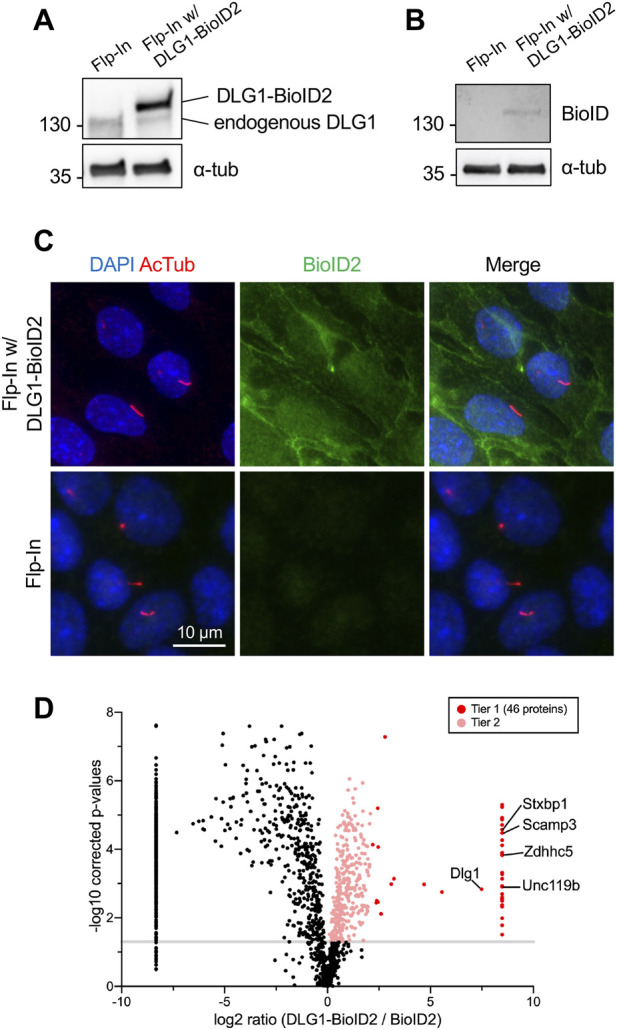
Validation of IMCD3 cells stably expressing rat DLG1-BioID2. **(A,B)** Western blots of IMCD3 Flp-In cell lines without (Flp-In) or with stable expression of rat DLG1-BioID2. Blots were probed with antibodies against DLG1 **(A)** or BioID2 **(B)** as well as α-tubulin (α-tub), which serves as loading control. Molecular mass markers are shown in kDa to the left of the blots. **(C)** IFM analysis of the indicated IMCD3 Flp-In cell lines following 48 h of culture in supplement-free medium and staining with antibodies against acetylated α-tubulin (AcTub; red), which marks the cilium, and BioID2 (green). Nuclei were stained with DAPI. **(D)** Volcano plot displaying differential protein interaction between DLG1-BioID2 and BioID alone. Logarithmic ratios are plotted against the negative logarithmic P-value of a Student’s t-test. Proteins are coloured according to their significance tier, and selected Tier 1 candidates are indicated. Proteins with infinite logarithmic ratios (median value in one set equal to 0) were forced to be one order of magnitude above or below the maximum or minimum ratios respectively.

To investigate the mechanism by which DLG1 regulates ciliary composition, we sought to determine its interactome in ciliated IMCD3 cells. To this end, we established an IMCD3 Flp-In cell line stably expressing DLG1 fused to BioID2 at its C-terminus (DLG1-BioID2) and induced ciliogenesis by growing the cells in supplement-free medium for 48 h. Western blotting and immunofluorescence microscopy (IFM) analysis with anti-DLG1 or -BioID2 antibodies confirmed the successful expression of DLG1-BioID2 fusion protein in these cells (Figures 1A–C), and IFM staining for the cilia marker acetylated α-tubulin confirmed their ciliation status (Figure 1C). Following incubation with biotin to induce BioID2-mediated proximity labeling, biotinylated proteins were identified by mass spectrometry (MS) and quantified against the negative control. As a negative control, we used our previously described IMCD3 Flp-In line expressing BioID2 alone (Aslanyan et al., 2023; Rezi et al., 2024). Our mass spectrometry analysis yielded approximately 2100 proteins, of which we have categorized the significantly enriched proteins from the DLG1-BioID2 dataset using a 2-tier stringency approach. Focusing on the Tier 1 proteins (q-value ≤0.05 and Significance A ≤ 0.05), we identified 46 high-confidence, putative DLG1 interactors, including several well-known DLG1 partners such as MPP7 and LIN7C (Leonoudakis et al., 2004; Stucke et al., 2007; Bohl et al., 2007; Yang et al., 2010), GPSM2 (Saadaoui et al., 2014), LRRC1 (Saito et al., 2001), VANGL1 (Sundell et al., 2018) and DVL1 (Liu et al., 2016), supporting the validity of our approach (Figure 1D; Table 2; Supplementary Table S2). In addition, CXADR, another candidate identified in our screen, was independently reported as a DLG1 proximity interactor in a separate study (Go et al., 2021). To address the relevance of our DLG1-BioID2 dataset in a ciliary context, we systematically cross-referenced the 46 candidate DLG1 interactors with published ciliary/centrosomal proteome/interactome datasets (Liu et al., 2007; Mick et al., 2015; Gupta et al., 2015; Datta et al., 2015; Sim et al., 2020; Karunakaran et al., 2020; Masek et al., 2022; Rezi et al., 2024; Liu et al., 2024; Loukil et al., 2025; Hansen et al., 2025). This analysis revealed supporting evidence for ciliary or centrosomal localization for 35 out of the 46 candidate DLG1 interactors (Table 2; Supplementary Table S2), underscoring the strong ciliary relevance of the identified interactome.

**TABLE 2:** List of DLG1 interactors identified by proximity-labeling proteomics in mouse IMCD3 cells stably expressing rat DLG1-BioID2 or BioID2 alone (negative control).

Uniprot ID	Gene/Protein name	DLG1-BIOID2:BIOID2 ratio	Ciliary evidence
P27773	Pdia3	8.48	Found in multiple ciliary proteomes (Hansen et al., 2025; Rezi et al., 2024). Enriched in *Bbs1* mutant zebrafish photoreceptor OSs (Masek et al., 2022).
Q91VS8	Farp2	8.48	Tier 3 ciliary candidate in (Rezi et al., 2024). Enriched in cilia of *Ift27* KO IMCD3 cells (Mick et al., 2015).
Q8BHD8	Pcmtd2	8.48	Tier 1 ciliary candidate in (Rezi et al., 2024).
Q8C4B4	Unc119b	8.48	Found in multiple ciliary proteomes (Hansen et al., 2025; Rezi et al., 2024). Enriched in *Lztfl1* mutant photoreceptor OSs (Datta et al., 2015). Targets myristoylated proteins to cilia (Wright et al., 2011).
O35609	Scamp3	8.48	Tier 3 ciliary candidate in (Rezi et al., 2024).
Q9CQB2	Fam195a	8.48	Tier 3 ciliary candidate in (Rezi et al., 2024).
Q9DB42	Znf593	8.48	Tier 3 ciliary candidate in (Rezi et al., 2024).
B2RRE7	Otud4	8.48	​
O08599	**Stxbp1**	8.48	Found in cilia proteomes of mouse NIH3T3 cells (Liu et al., 2024) and neurons (Loukil et al., 2025); enriched in *Lztfl1* mutant mouse and zebrafish *Bbs1* mutant photoreceptor OSs (Datta et al., 2015; Masek et al., 2022).
P0CG14	Chtf8	8.48	​
P16125; P00342	Ldhb	8.48	Found in multiple ciliary proteomes (Hansen et al., 2025). Enriched in *Lztfl1* mutant photoreceptor OSs (Datta et al., 2015).
P18608	Hmgn1	8.48	CEP290 interactor in (Gupta et al., 2015).
P35601	Rfc1	8.48	​
P51612	Xpc	8.48	CETN2 interactor in (Karunakaran et al., 2020).
P58059	Mrps21	8.48	​
P59326	Ythdf1	8.48	CEP135 interactor in (Gupta et al., 2015). Found in mouse photoreceptor sensory cilium proteome (Liu et al., 2007).
P62915	Gtf2b	8.48	​
P63158	Hmgb1	8.48	Tier 2 ciliary candidate in (Rezi et al., 2024).
Q00288	Pax8	8.48	Tier 1 ciliary candidate in (Mick et al., 2015).
Q3THE2; Q9CQ19	Myl12b	8.48	Found in multiple ciliary proteomes (Hansen et al., 2025).
Q80W68	Kirrel1	8.48	Found in multiple ciliary proteomes (Hansen et al., 2025).
Q8BI84	Mia3	8.48	Found in multiple ciliary proteomes (Hansen et al., 2025).
Q8BJU0	Sgta	8.48	​
Q8BP71	Rbfox2	8.48	DNAH5 interactor in (Karunakaran et al., 2020).
Q8BVD5	**Mpp7**	8.48	​
Q8VDU0	**Gpsm2**	8.48	Found in mouse photoreceptor sensory cilium proteome (Liu et al., 2007).
Q8VDZ4	**Zdhhc5**	8.48	Found in multiple ciliary proteomes (Hansen et al., 2025). Confirmed ciliary presence and function in mouse kidney epithelial cells (Rezi et al., 2025).
Q9CPV5	Pmf1	8.48	​
Q9CY94	Gins3	8.48	Found in ciliary proteome of *Xenopus* multiciliated cells (Sim et al., 2020).
Q9CYL5	Glipr2	8.48	Found in multiple ciliary proteomes (Hansen et al., 2025).
Q9D0J8	Ptms	8.48	​
Q9JKV1	Adrm1	8.48	​
Q9Z127	Slc7a5	8.48	Found in cilia proteome of mouse neurons (Loukil et al., 2025). Enriched in *Bbs1* mutant zebrafish photoreceptor OSs (Masek et al., 2022).
Q811D0; Q91XM9; P70175; Q62108	Dlg1	7.48	Found in multiple ciliary proteomes (Hansen et al., 2025). Enriched in *Lztfl1* mutant photoreceptor OSs (Datta et al., 2015). Confirmed ciliary function in mouse kidney epithelial cells (Rezi et al., 2024).
Q80VQ1	**Lrrc1**	5.56	Tier 3 ciliary candidate in (Rezi et al., 2024). Enriched in cilia of *Ift27* KO IMCD3 cells (Mick et al., 2015).
Q80Z96	**Vangl1**	4.69	Found in multiple ciliary proteomes (Hansen et al., 2025).
P51141	**Dvl1**	3.22	Found in multiple ciliary proteomes (Hansen et al., 2025). Confirmed localization at ciliary base (Simons et al., 2005).
Q9D3D9	Atp5d	3.10	Tier 3 ciliary candidate in (Rezi et al., 2024); enriched in *Lztfl1* mutant photoreceptor OSs (Datta et al., 2015).
O88291	Znf326	2.79	Tier 3 ciliary candidate in (Rezi et al., 2024).
Q9CRB9	Chchd3	2.60	Tier 3 ciliary candidate in (Rezi et al., 2024).
Q8K4G5	Ablim1	2.46	Found in multiple ciliary proteomes (Hansen et al., 2025).
P97792	Cxadr	2.45	Found in multiple ciliary proteomes (Hansen et al., 2025). Enriched in cilia of *Ift27* KO IMCD3 cells (Mick et al., 2015), and *Lztfl1* mutant mouse and zebrafish *Bbs1* mutant photoreceptor OSs (Datta et al., 2015; Masek et al., 2022).
E9QAT4	Sec16a	2.44	Found in multiple ciliary proteomes (Hansen et al., 2025).
Q05920	Pc	2.39	Found in multiple ciliary proteomes (Hansen et al., 2025).
O88952	**Lin7c**	2.37	Found in mouse photoreceptor sensory cilium proteome (Liu et al., 2007).
Q3ULD5	Mccc2	2.20	​

Proteins in bold were confirmed to bind to DLG1 previously or in this study (see text for details). See also Supplementary Table S2. OS: outer segment.

**FIGURE 2:**
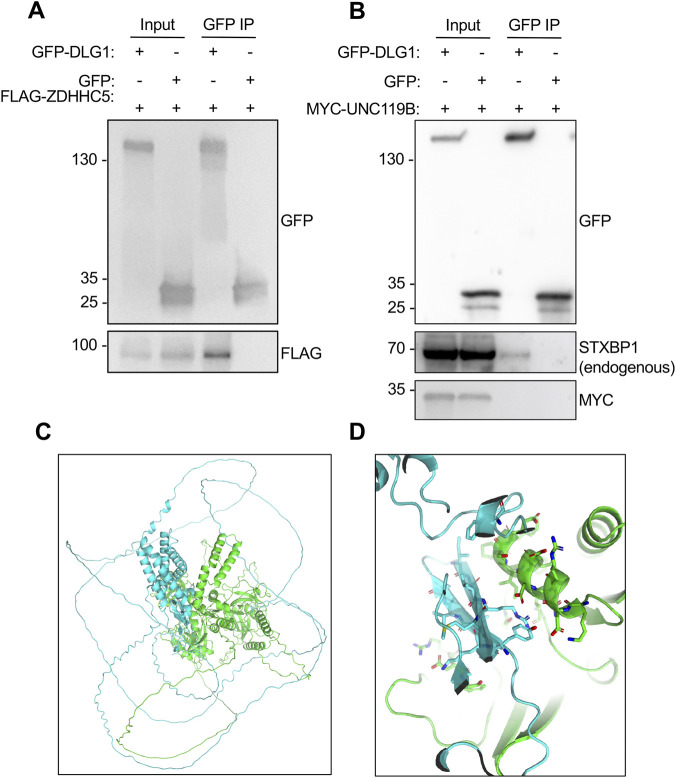
DLG1 binds to ZDHHC5 and STXBP1. **(A,B)** HEK293T cells co-expressing GFP-DLG1 or GFP alone (negative control) with FLAG-ZDHHC5 **(A)** or MYC-UNC119B **(B)** were subjected to IP with GFP beads, and input and IP pellets analysed by western blotting using antibodies as indicated. Molecular mass markers are shown in kDa to the left. **(C,D)** Structural model of the DLG1-ZDHHC5 interaction. **(C)** AlphaFold2-Multimer model showing the predicted structure of the complex between DLG1 (green) and ZDHHC5 shown in cartoon representation. In addition to the structured domains, large regions are predicted to be disordered. **(D)** Zoom-in on the interface between the GUK-like domain (residues 715–890) of DLG1 and ZDHHC5’s N-terminal region (residues 70–250). Interface residues with PAE < 4Å are highlighted as sticks.

To validate candidate ciliary/centrosomal interactors identified in the DLG1-BioID2 screen, we initially focused on ZDHHC5 and UNC119B, as previous studies have implicated both proteins in the regulation of ciliary length and/or protein content (Rezi et al., 2025; Wright et al., 2011; Stephen and Ismail, 2016). Notably, ZDHHC5 was also shown to interact physically and functionally with DLG4, a close homolog of DLG1, at neuronal synapses (Brigidi et al., 2015). We co-expressed GFP-DLG1 or GFP alone (negative control) with or without FLAG-ZDHHC5 or MYC-UNC119B in HEK293T cells and performed GFP immunoprecipitation (IP) assays followed by Western blotting with antibodies against GFP, MYC or FLAG. Our results demonstrated that GFP-DLG1 binds physically and specifically to FLAG-ZDHHC5 (Figure 2A), whereas no interaction with MYC-UNC119B was detected in this assay (Figure 2B).

In addition, the IP assays confirmed binding of GFP-DLG1 to two other candidates from our DLG1-BioID2 screen: STXBP1 (Figure 2B) and SCAMP3 (Supplementary Figure S1). In support of the latter result, SCAMP3 also interacted with GFP-KIF13B (Supplementary Figure S1), a well-described direct interactor of DLG1 and DLG4 (Asaba et al., 2003; Zhu et al., 2016). Collectively, these data validate physical interactions between DLG1 and three of the four tested candidate interactors (STXBP1, ZDHHC5 and SCAMP3) from our DLG1-BioID2 screen. While physical interaction between DLG1 and UNC119B could not be confirmed in our IP assay, we cannot exclude the possibility that the N-terminal MYC tag on MYC-UNC119B interferes with binding to DLG1.

### 3.2 Structural modeling of the DLG1-ZDHHC5 interaction

We next assessed the structural basis of the physical interaction between DLG1 and ZDHHC5, STXBP1 and SCAMP3. We performed structural modeling of DLG1 with each of these interactors using AlphaFold2-Multimer v3 (Evans et al., 2022). While this approach did not predict high-confidence direct interactions between DLG1 and either STXBP1 or SCAMP3, it identified a likely direct interaction between DLG1 and ZDHHC5 (Figures 2C,D). The predicted model includes full-length DLG1 (905 residues) comprising six domains: L27 (residues 4–64), three PDZ domains (PDZ-1: 224-311, PDZ-2: 319-406, PDZ-3: 466–547), an SH3 domain (581–651), and a C-terminal GUK-like domain (715–890). ZDHHC5 has 715 residues and contains an N-terminal regulatory region, a central DHHC catalytic domain (104–154), and an extensive intrinsically disordered C-terminal region (289-715, 60% of the protein). Notably, the predicted interaction involves exclusively well-structured regions: The GUK-like domain of DLG1 (21 interface residues: 725-727, 729-737, 740, 742, 759-761, 800, 806–808) and the N-terminal regulatory region of ZDHHC5 upstream of the catalytic domain (12 interface residues: 91-95, 99-103, 225) (Figures 2C,D).

The predicted DLG1-ZDHHC5 complex features a mixed hydrophobic/hydrophilic interface with a mean pLDDT score of 70.2 and a mean Predicted Aligned Error (PAE) of 5.3Å across interface residues (Supplementary Figure S2). Interface residues were defined using a dual criterion: PAE < 4Å and inter-residue atomic distance ≤ 8Å between residue pairs. Using these thresholds, we identified 61 high-confidence inter-chain contacts involving 33 unique residues: 21 from DLG1 (residues 725-727, 729-737, 740, 742, 759-761, 800, 806-808) and 12 from ZDHHC5 (residues 91-95, 99-103, 225). The highest-confidence contact was a predicted salt-bridge between DLG1 Asp730 and ZDHHC5 Arg102 with a PAE of 3.1Å. Together with an ipSAE (interface predicted Score from Aligned Errors) of 0.409, these metrics indicate moderate confidence in the predicted structure of the DLG1-ZDHHC5 interaction (Dunbrack, 2025).

The structural model suggests that DLG1 binds to ZDHHC5’s regulatory region rather than its catalytic DHHC domain (residues 104–154), which shows minimal involvement in the interface. This positioning is consistent with a regulatory or scaffolding role, where DLG1 may modulate ZDHHC5 activity allosterically or recruit it to specific substrates or cellular locations. The moderate structural confidence suggests the interaction may be transient or context-dependent, consistent with a regulatory interaction rather than a constitutive stable complex. These results are in line with previous work indicating activity-regulated interaction between ZDHHC5 and DLG4 at the neuronal synapse (Brigidi et al., 2015).

### 3.3 Effect of DLG1 loss on ciliary localization of ZDHHC5 and STXBP1

**FIGURE 3:**
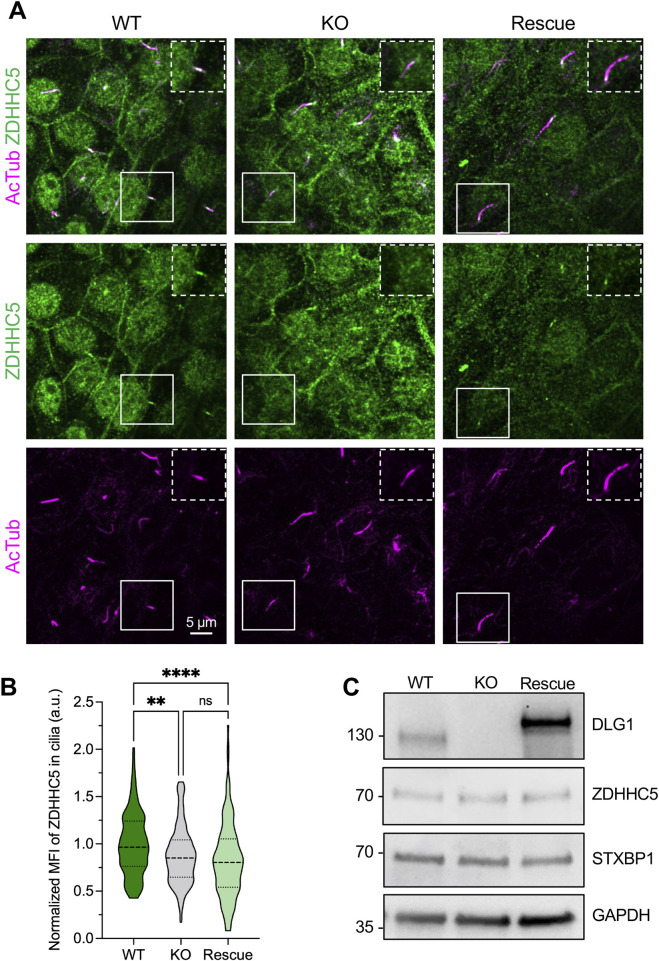
Ciliary localization of ZDHHC5. **(A)** IFM analysis of ciliary localization of ZDHHC5 in control (WT) and *Dlg1* knockout cells without (KO) or with (Rescue) stable re-expression of mCherry-DLG1. Cells were cultured for 24 h in supplement-free medium and subjected to IFM analysis using antibodies against ZDHHC5 (green) and acetylated α-tubulin (AcTub; magenta) to stain the ciliary axoneme. Insets show zoom-ins of the cilium-basal body axis. Note that average ciliary length is unaffected by DLG1 loss under these conditions (Rezi et al., 2024). **(B)** Quantification of mean fluorescence intensity (MFI) of ZDHHC5 in cilia of the indicated cell lines, based on images as in **A. (C)** Western blot analysis of whole cell lysate from the indicated cell lines using antibodies listed to the right of the blots. Molecular mass markers are shown in kDa to the left. GAPDH serves as loading control.

ZDHHC5 is a palmitoyl transferase that mainly localizes to the plasma membrane and plays an important role in a broad range of cellular processes across multiple cell types, including cardiac function, synaptic plasticity, cell adhesion, fatty acid uptake, host–pathogen interactions, and immune signaling (Woodley and Collins, 2021). We previously showed that ZDHHC5 also localizes to primary cilia of different mouse kidney epithelial cell lines (mCCD, IMCD3), where it accumulates upon BBSome dysfunction and regulates both ciliary length and Polycystin-2 levels (Rezi et al., 2025). These phenotypes closely resemble those observed in *Dlg1* KO cells, although the effect of DLG1 loss on ciliary length was only observed in fully polarized kidney epithelial cells (e.g., filter-grown mCCD cells) and not IMCD3 cells grown in standard 2D culture conditions (Rezi et al., 2024). Given our structural and biochemical data suggesting that DLG1 directly interacts with ZDHHC5 (Figures 2A,C,D; Supplementary Figures S2, S3), we investigated whether DLG1 regulates the ciliary localization of ZDHHC5. Specifically, we performed IFM analysis in our previously characterized *Dlg1* KO IMCD3 cells as well as control (wild type; WT) and mCherry-DLG1-expressing rescue lines (Rezi et al., 2024). Following 24 h of incubation in supplement-free medium to induce ciliogenesis, cells were co-stained for acetylated α-tubulin (ciliary marker) and ZDHHC5 and imaged by confocal microscopy. Consistent with our earlier findings (Rezi et al., 2025), ZDHHC5 localized to the proximal region of cilia in IMCD3 cells (Figure 3A). Notably, loss of DLG1 resulted in a significant reduction of ciliary ZDHHC5 localization. However, this phenotype was not rescued by stable expression of mCherry-DLG1 (Figures 3A,B), indicating that off-targets of *Dlg1* KO or additional regulatory mechanisms cannot be ruled out in this context; it is also possible that the mCherry tag on the DLG1 rescue construct interferes with its ability to regulate ZDHHC5 localization. Quantitative Western blot analysis revealed comparable total cellular ZDHHC5 protein levels in WT, *Dlg1* KO and rescue cell lines (Figure 3C; Supplementary Figure S3), indicating that DLG1 does not affect ZDHHC5 protein abundance.

**FIGURE 4:**
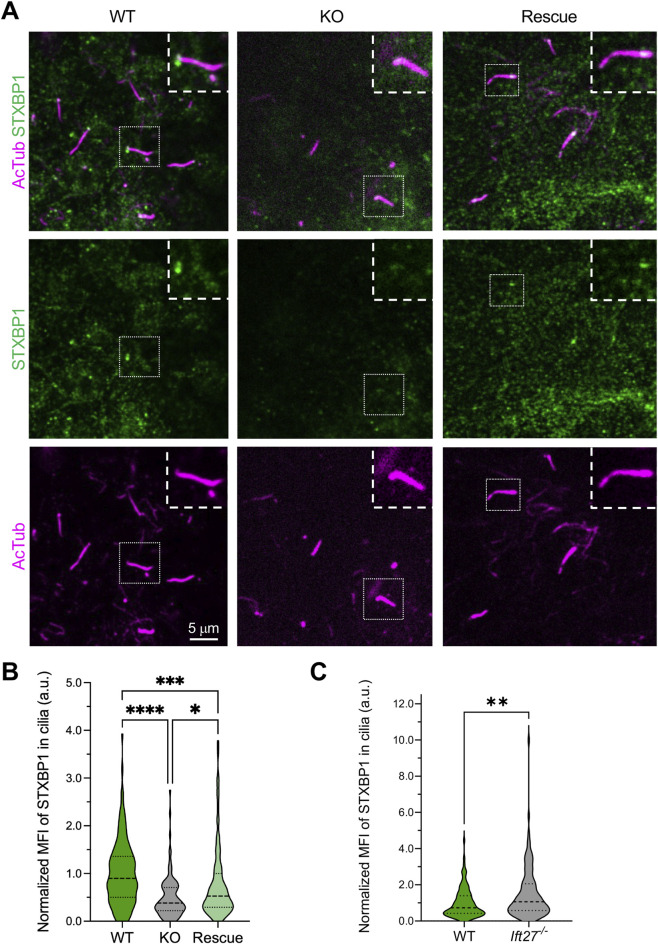
STXBP1 localizes to the base of primary cilia in a DLG1-dependent manner. **(A)** IFM analysis shows ciliary base localization of STXBP1 in control (WT) IMCD3 cells, which is lost in *Dlg1* KO cells, and restored upon stable expression of mCherry-DLG1 in the KO line (Rescue). Cells were serum-starved for 24 h and subjected to IFM analysis using antibodies against STXBP1 (green) and acetylated α-tubulin (AcTub; magenta), which stains the ciliary axoneme. Insets show zoom-ins of the cilium-basal body axis. **(B)** Quantification of MFI of STXBP1 at the ciliary base of the indicated cell lines, based on images as in **A** (N = 3; WT n = 143, KO n = 107, Rescue n = 98). **(C)** Quantification of MFI of STXBP1 along the cilia of the indicated cell lines, based on images as in Supplementary Figure S4. (N = 3; WT n = 108, *Ift27* ^*−/−*^ n = 106).

STXBP1, also known as Munc18-1, is best known for its key role in organizing the neuronal SNARE complex that drives synaptic vesicle fusion (Sudhof and Rothman, 2009; Jahn and Fasshauer, 2012). The importance of STXBP1 in neuronal function is emphasized by the high prevalence of neuronal disorders associated with pathogenic variants in the corresponding gene (Xian et al., 2022). While ciliary functions for STXBP1 have not been extensively characterized, STXBP1 was detected in several independent ciliary proteomic datasets, including those from mouse NIH3T3 cells (Liu et al., 2024), neurons (Loukil et al., 2025), and photoreceptor outer segments (OSs) (Datta et al., 2015). Notably, STXBP1 accumulated aberrantly, along with DLG1, in the photoreceptor OS of an *Lztfl1* mutant that exhibits defective BBSome-mediated retrieval of ciliary proteins (Datta et al., 2015). Consistent with this, STXBP1 was also shown to accumulate in the photoreceptor OS of a zebrafish *bbs1* mutant (Masek et al., 2022). More recently, a preprint showed localization of both STXBP1 and DLG1 to primary cilia of cultured primary rat embryonic striatal neurons and motile cilia of the *Xenopus laevis* embryonic epidermis (Kostyanovskaya et al., 2024). Given that STXBP1 interacted with DLG1 in our BioID2 screen (Table 2) and was confirmed by IP assay (Figure 2B), we tested whether STXBP1 localizes to cilia in IMCD3 cells and whether its localization depends on DLG1. To this end, we performed IFM analysis in ciliated WT, *Dlg1* KO and rescue IMCD3 cell lines (Rezi et al., 2024) using antibodies against endogenous STXBP1 and acetylated α-tubulin (ciliary marker). Our results indicated that STXBP1 is concentrated at the ciliary base of WT cells, in line with previous work (Datta et al., 2015; Masek et al., 2022; Loukil et al., 2025; Kostyanovskaya et al., 2024). Loss of *Dlg1* significantly reduced STXBP1 localization at this site, a phenotype that was partially rescued by re-expression of mCherry-DLG1 (Figures 4A,B). Quantitative Western blot analysis indicated that total cellular STXBP1 protein levels are comparable in WT, *Dlg1* KO and rescue cell lines (Figure 3C; Supplementary Figure S3). Furthermore, loss of IFT27 significantly upregulated STXBP1 ciliary localization in IMCD3 cells (Figure 4C; Supplementary Figure S4). Taken together, we conclude that ZDHHC5 and STXBP1 localize to the base of cilia in IMCD3 cells and that DLG1 is required for ciliary recruitment or maintenance of STXBP1, whereas its role in ZDHHC5 localization remains unresolved.

### 3.4 Depletion of *zdhhc5* in zebrafish causes elongated cilia and ciliopathy-like phenotypes

In a recent study we showed that *Zdhhc5* KO in mCCD cells leads to abnormally long primary cilia and reduced ciliary Polycystin-2 content (Rezi et al., 2025). In addition, a *Zdhhc5* KO mouse model exhibited phenotypes overlapping with those of some ciliopathies (Reiter and Leroux, 2017; Mill, Christensen, and Pedersen, 2023), including short snout, reduced retinal thickness and fat tissue content, cardiac defects and unilateral hydronephrosis (da Silva-Buttkus et al., 2023). It is currently unknown whether some of these reported phenotypes are linked to ciliary defects, although a recent study indicated that loss of ZDHHC5 in the mouse causes male infertility and sperm tail malformations (Wang et al., 2025).

**FIGURE 5:**
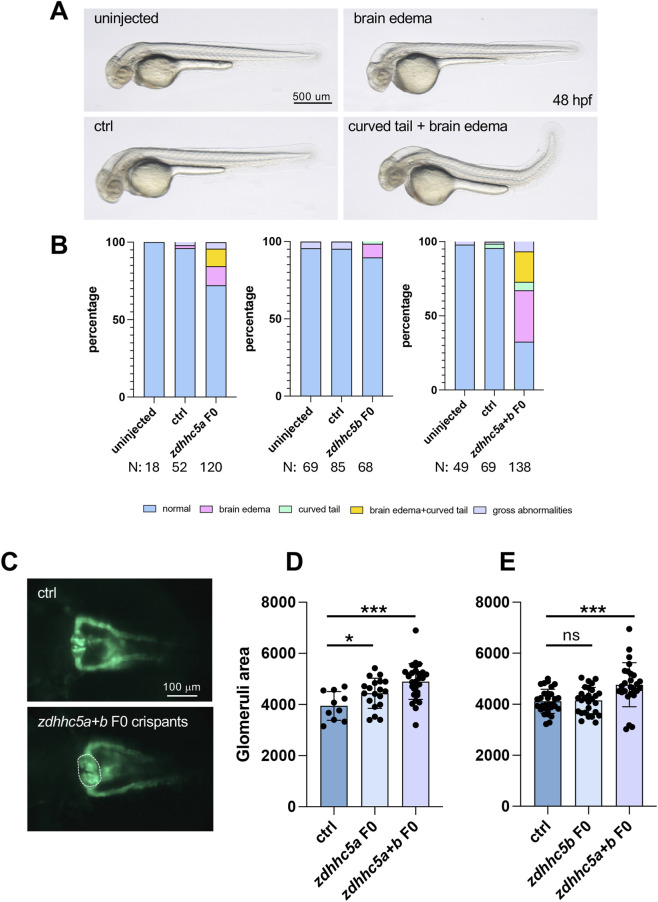
Phenotypic assessment of zebrafish *zdhhc5a*, *zdhhc5b* and *zdhhc5a + b* F0 crispants. **(A)** Representative images of gross morphology in F0 crispants at 48 h post fertilization (hpf). **(B)** Quantification of observed gross morphological phenotypes. **(C)** Representative images of pronephros from control (ctrl) and *zdhhc5a + b* RNPs injected *Tg(wt1b:GFP)* zebrafish larvae at 48 hpf. **(D)** Quantification of glomerular area in ctrl, *zdhhc5a* or *zdhhc5a + b* RNPs injected F0 crispants. **(E)** Quantification of glomerular area in ctrl, *zdhhc5b or zdhhc5a + b* RNPs injected F0 crispants.

To address potential cilia-related functions of ZDHHC5 *in vivo*, we used the zebrafish model and generated *zdhhc5* F0 crispants to mimic biallelic null mutants. Gross morphological analysis revealed that larvae targeted for either *zdhhc5* isoforms displayed brain edema and curved tails - phenotypes frequently associated with ciliary dysfunction - and these defects were more prominent when both isoforms were simultaneously disrupted (Figures 5A,B). To investigate kidney phenotypes, we targeted *zdhhc5a*, *zdhhc5b*, or both isoforms in the *Tg(wt1b:GFP)* background. Combined depletion of *zdhhc5a* and *zdhhc5b* caused significant glomerular enlargement, indicating that Zdhhc5 is required for maintaining pronephric morphology (Figures 5C–E). Targeting *zdhhc5a* alone also enlarged the glomeruli, whereas targeting *zdhhc5b* alone produced no detectable abnormalities, suggesting that *zdhhc5a* plays a dominant or isoform-specific role in glomerular development. The presence of cysts predominantly in double-crispants further implies partial functional redundancy between the isoforms.

**FIGURE 6:**
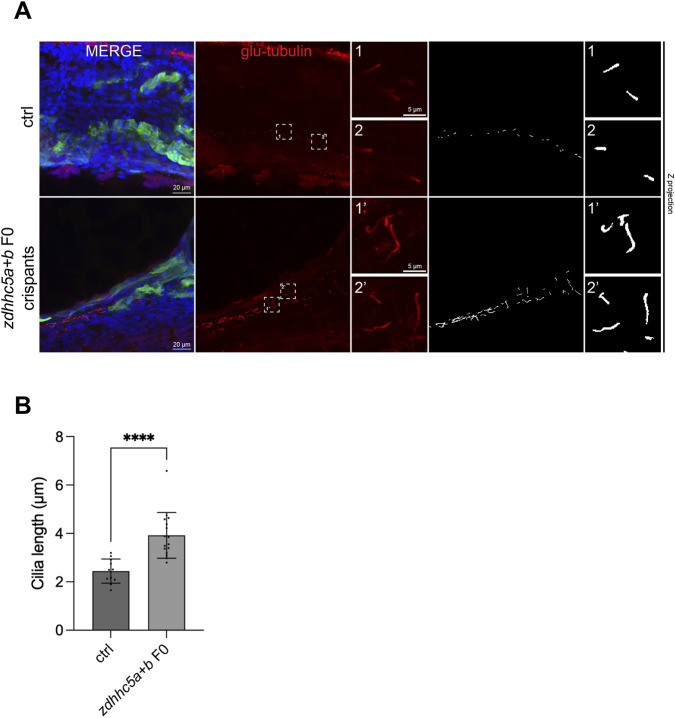
Depletion of *zdhhc5* isoforms in zebrafish causes ciliary elongation in the pronephros. **(A)** Confocal images of the posterior pronephric duct showing elongated cilia in *zdhhc5a + b* depleted F0 crispants compared to ctrl RNPs injected *Tg(wt1b:GFP)* zebrafish. **(B)** Comparison of the mean ciliary length between ctrl and F0 crispant fish. ****, p < 0.0001 using unpaired *t*-test. Based on data from 11 fish for ctrl and 16 fish for *zdhhc5a + b* F0 crispants in 2 independent experiments.

To determine whether the morphological kidney defects were accompanied by alterations in ciliary structure, we next examined ciliary length in the posterior pronephric duct of *zdhhc5a + b* F0 crispants, a region in which individual motile cilia can be readily visualized. Quantitation of confocal images revealed a significant increase in ciliary length in *zdhhc5a + b*-depleted larvae compared with scramble RNP-injected controls (Figures 6A,B). This ciliary elongation phenotype was recapitulated in brain (Supplementary Figure S5B,C) and is consistent with our cell-culture findings, in which loss of ZDHHC5 led to increased ciliary length in mCCD cells (Rezi et al., 2025). Thus, these *in vivo* results further support a conserved role for Zdhhc5 in regulating ciliary morphology within the pronephros as well as brain.

## 4 Discussion

To sum up, our study identifies and validates new physical interactors of DLG1 that provide new mechanistic insight into its role in regulating primary cilia composition and possibly length. Using BioID2 proximity labeling in ciliated IMCD3 cells, we identified 46 high-confidence DLG1 interactors, many of which are known ciliary or centrosomal proteins. Follow-up co-IP experiments confirmed binding between DLG1 and three candidates—ZDHHC5, STXBP1, and SCAMP3—while interaction with UNC119B was not detected.

Structural modeling further predicted a likely direct interaction specifically between the GUK-like domain of DLG1 and the regulatory N-terminus of ZDHHC5. While loss of DLG1 disrupted the ciliary localization of ZDHHC5 and STXBP1, rescue of ZDHHC5 localization with exogenously expressed mCherry-DLG1 was not observed making it uncertain whether DLG1 is strictly required for ciliary localization of ZDHHC5. Instead, DLG1 may modulate ZDHHC5 activity at the cilium, consistent with its predicted direct binding to the regulatory N-terminal domain of ZDHHC5.

In our previous work, we showed that depletion of either DLG1 or ZDHHC5 in cultured mCCD cells caused ciliary elongation and reduced ciliary presence of Polycystin-2 (Rezi et al., 2024; Rezi et al., 2025), suggesting that these proteins may interact functionally in this context. The mechanisms that control ciliary Polycystin-2 localization are complex and multifactorial (Hu and Harris, 2020), but amongst other factors, ciliary presence of Polycystin-2 was reported to rely on its interaction with Polycystin-1 (Freedman et al., 2013; Kim et al., 2014). In addition, ciliary localization of Polycystin-1 depends on its palmitoylation (Roy and Marin, 2018), indicating that ZDHHC5 potentially promotes ciliary trafficking of Polycystin-2 through palmitoylation of Polycystin-1. Although further experimental validation is required to test this idea, it is notable that ZDHHC5, DLG1 as well as STXBP1 and SCAMP3 were identified as putative Polycystin-1 interactors in two independent studies (Lin et al., 2023; Lukmani et al., 2025), supporting a possible role for these proteins in regulating ciliary Polycystin-1 trafficking. Moreover, since ablation of Polycystin-1 or Polycystin-2 was reported to increase ciliary length in kidney epithelial cells (Liu et al., 2018), defective ciliary trafficking of these proteins might contribute to the increased ciliary length phenotype observed upon loss of DLG1 (Rezi et al., 2024) or ZDHHC5 ((Rezi et al., 2025); this study).

While cell-based work supports a role for DLG1 and ZDHHC5 in regulating ciliary composition and/or length, the extent to which ciliary dysfunction contributes to the phenotypes observed following loss of these genes *in vivo* remains unclear. In the case of DLG1, gene-trap mutant mice exhibit neonatal lethality, growth retardation, craniofacial malformations, and renal hypoplasia characterized by impaired ureteric bud branching and reduced nephron formation (Caruana and Bernstein, 2001; Naim et al., 2005). Complete KO of *Dlg1* results in perinatal death due to profound cardiovascular and craniofacial defects, as well as abnormal urogenital development (Mahoney et al., 2006; Iizuka-Kogo et al., 2007). Histological analyses further reveal that KO animals lack stromal cells between the urothelium and smooth muscle layers, and circular smooth muscle cells in the ureter are misaligned, leading to defective peristalsis and hydronephrosis (Mahoney et al., 2006). Conditional deletion of *Dlg1* in the metanephric mesenchyme-the progenitor population giving rise to nephron segments, results in kidneys with glomerular cysts, dilated proximal tubules, proteinaceous casts, and widespread inflammation, accompanied by elevated blood urea nitrogen and serum creatinine levels, indicative of renal failure (Ahn et al., 2013).

In humans, DLG1 is deleted in the 3q29 microdeletion syndrome, which is linked to intellectual disability, craniofacial dysmorphisms, and additional phenotypes including microcephaly, cleft lip/palate, horseshoe kidney, and hypospadias (Willatt et al., 2005). Moreover, DLG1 has been implicated as a susceptibility gene for CAKUT (Westland et al., 2015), and a missense variant (p.T489R) was indeed identified in a patient with CAKUT (Nicolaou et al., 2016). Our previous study indicated that this variant impairs the ability of DLG1 to regulate ciliary protein content (Rezi et al., 2024), suggesting that dysfunctional cilia may at least in part contribute to the CAKUT disease etiology.

Similar to DLG1, ZDHHC5 is broadly expressed across vertebrate tissues and participates in diverse cellular functions, including modulation of synaptic plasticity, regulation of heart physiology, control of cell–cell adhesion, and facilitation of fatty-acid uptake (Woodley and Collins, 2021). Mice lacking ZDHHC5 display several developmental and physiological abnormalities, such as a shortened snout, decreased retinal thickness, reduced adipose tissue, electrocardiogram anomalies, and unilateral hydronephrosis (da Silva-Buttkus et al., 2023). In addition, ZDHHC5 loss was also associated with defects in spermatogenesis and sperm motility (Wang et al., 2025). While some of these features overlap with characteristic manifestations of ciliopathies (Reiter and Leroux, 2017; Mill et al., 2023), it remains unclear to what extent the phenotypes observed in ZDHHC5-deficient mice are directly attributable to ciliary dysfunction. However, we found that in zebrafish, CRISPR-mediated depletion of *zdhhc5* isoforms produced phenotypes commonly reported in ciliopathy models, including brain edema, curved tails, pronephric defects, and significantly elongated cilia in the brain and pronephros. While these features are not specific to ciliopathies and may reflect broader developmental disturbances, the coexistence of ciliary abnormalities with kidney phenotypes suggests that impaired ciliary function may be a contributing factor. Further studies are needed to establish a direct causal link between ciliary defects and the observed morphological changes. Nevertheless, the presence of ciliary abnormalities supports a role for ZDHHC5 in the regulation of ciliary structure and function.

Several candidate or validated DLG1 interactors identified in our study were previously shown to accumulate in cilia of BBSome-deficient cells. These include the known/confirmed DLG1 interactors ZDHHC5 (Rezi et al., 2025), STXBP1 (Datta et al., 2015; Masek et al., 2022), and LRRC1 (Saito et al., 2001; Mick et al., 2015), but also candidate interactors PDIA3, FARP2, UNC119B, LDHB, SLC7A5, ATP5D, and CXADR (Table 2) (Datta et al., 2015; Mick et al., 2015; Masek et al., 2022). In addition, DLG1 itself was shown to accumulate in photoreceptor OSs of *Lztfl1* mutant mice (Datta et al., 2015), which are characterized by defective BBSome-mediated protein retrieval from cilia (Datta et al., 2015; Wingfield et al., 2018). Collectively, these findings support the existence of a shared, BBSome-dependent mechanism that normally limits the ciliary abundance of DLG1 and its associated protein network.

The BBSome is known to function as a membrane protein cargo adapter during retrograde IFT (Lechtreck et al., 2009). It was shown to bind to its ciliary cargoes through an adapter protein, TOM1L2, which is an ESCRT-associated ubiquitin reader that recognizes polyubiquitinated cargoes such as specific GPCRs, mediating their retrieval from cilia (Shinde et al., 2023). It is unclear whether a similar mechanism downregulates ciliary levels of DLG1 and its interactors within this compartment. However, a study showed that the L27 domain in an N-terminal isoform of DLG4 (an L27+ splice form, also present in DLG1) directly binds to another ubiquitin reader, the ESCRT-0 protein HGS, which is involved in early endosomal sorting (Chetkovich et al., 2002). Although HGS was not detected in our DLG1-BioID2 screen, we identified SCAMP3 as a DLG1 interactor; SCAMP3 was previously shown to bind HGS in the context of epidermal growth factor receptor (EGFR) degradation and recycling (Aoh et al., 2009). Similarly, ubiquitinated STXBP1 was shown to bind directly to the ubiquitin-interacting motif of HGS (Pridgeon et al., 2009). Notably, we and others have shown that HGS localizes to primary cilia in mouse kidney epithelial cells where its ciliary presence and release in small extracellular vesicles are regulated by the BBSome and KIF13B (Volz et al., 2021; Rezi et al., 2025). KIF13B is a well-established DLG1 interactor (Asaba et al., 2003; Zhu et al., 2016) that also associated with SCAMP3 in our IP assays. Moreover, studies in *C. elegans* male sensory neurons demonstrated a role for HGS in down-regulating ciliary polycystins by promoting their endocytic retrieval and lysosomal degradation (Hu et al., 2007). It will therefore be interesting to explore if HGS plays a role in mediating retrieval of DLG1 and its interactors from cilia, and if so, whether the BBSome may be involved in this process.

Taken together, our study provides mechanistic insight that may help link ciliopathies and congenital kidney disorders such as CAKUT through a shared pathway involving ciliary regulation. Primary cilia play a key role in kidney development and signaling, and disruption of ciliary structure or protein composition is a known driver of both ciliopathies and developmental kidney anomalies. The identification of DLG1 and ZDHHC5 as potential regulators of ciliary length, composition, and protein trafficking suggests a mechanism by which their dysfunction could alter ciliary signaling and contribute to abnormal kidney morphogenesis. Given that DLG1 has already been implicated in human CAKUT and that polycystin localization is critical for renal function, these findings support the idea that defects in ciliary trafficking and membrane regulation may represent a shared underlying mechanism across a spectrum of kidney diseases. While further validation in human systems is needed, these findings suggest that the DLG1-ZDHHC5 axis may represent a candidate pathway for further exploration in future genetic and functional studies of kidney disease.

### 4.1 Limitations of our study

The current data suggesting ciliary functions for ZDHHC5 and DLG1 are largely derived from cell-based systems and animal models, and direct human functional evidence remains lacking. While structural modeling and IP assays support a direct interaction between DLG1 and ZDHHC5, further work is required to validate this experimentally. The reported *zdhhc5* crispant zebrafish phenotypes are primarily morphological and may reflect underlying developmental defects; more detailed functional characterization, particularly of kidney function *in vivo*, would be valuable. The use of stable zebrafish knockout lines could also help clarify the consistency and specificity of these observations. Overall, although the findings provide important mechanistic insights, their direct clinical relevance requires further evidence.

## 5 Resource identification initiative

To take part in the Resource Identification Initiative, please use the corresponding catalog number and RRID in your current manuscript. For more information about the project and for steps on how to search for an RRID, please click here.

## Data Availability

The mass spectrometry data for this study has been deposited at the MassIVE repository as MSV000101937. Any additional information required to reanalyze the data reported in this paper is available from the lead contact upon request.

## Supplementary material

Supplementary Figures Supplementary Figures 1‐5

Supplementary Table S1 Oligonucleotide primers used for *zdhhc5a* and *zdhhc5b* PCR in zebrafish.

Supplementary Table S2 Mass spectrometry data.
