# Supplementary material for: ZDHHC5 interacts physically and functionally with DLG1 at primary cilia and regulates ciliary length and kidney morphology
Source: Front Cell Dev Biol. 2026 Jun 10;14:1805468. doi: 10.3389/fcell.2026.1805468 (PMC13290907; doi:10.3389/fcell.2026.1805468)
Supplement: Supplementary file 2 [file DataSheet1.pdf]

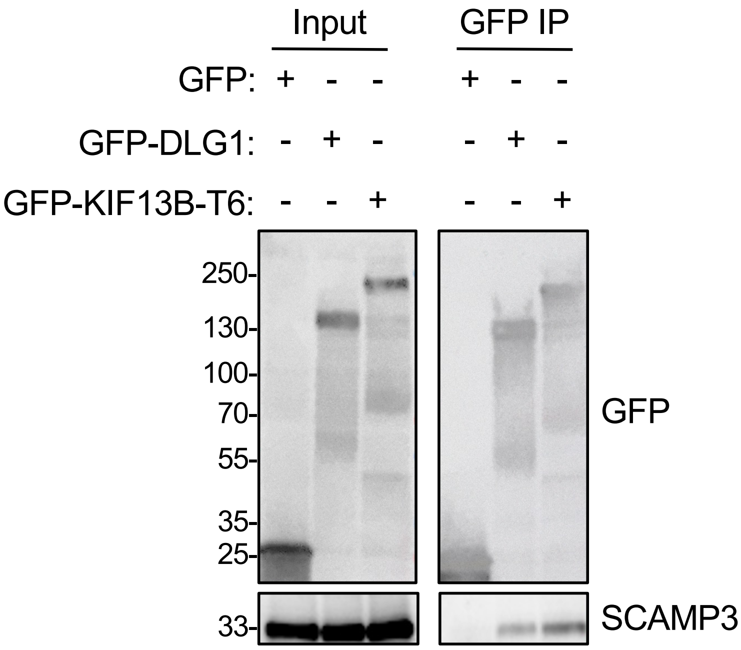

Figure S1. DLG1 and KIF13B bind to SCAMP3. Related to Figure 2. HEK293T cells expressing GFP alone (negative control), GFP-DLG1 or GFP-KIF13B-Tail 6 (GFP-KIF13B-T6; residues 561-1,826 (Schou et al. 2017)) were subjected to IP with GFP beads, and input and IP pellets were analyzed by SDS-PAGE and western blotting using antibodies as indicated. Molecular mass markers are shown in kDa to the left.

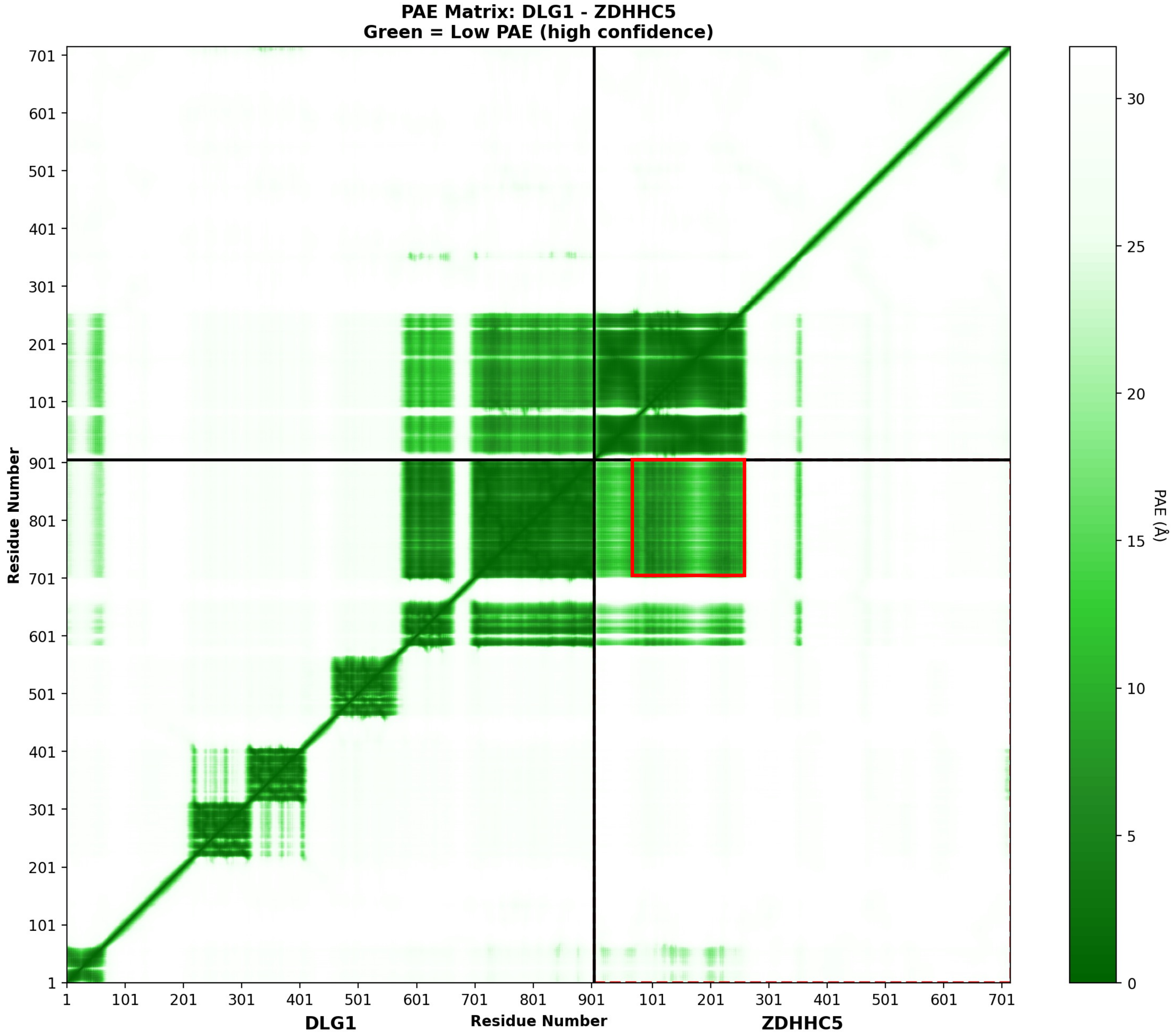

Figure S2. Predicted aligned error for the DLG1-ZDHHC5 complex. Predicted Aligned Error (PAE) matrix for the DLG1-ZDHHC5 complex. Related to Figure 2. Colored from dark green (low PAE, high confidence) to white (high PAE, low confidence). The box highlights the interface region between DLG1's GUK-like domain and ZDHHC5's N-terminal region. High PAE values in the lower-right correspond to ZDHHC5's intrinsically disordered C-terminus (residues 289-715). The box highlights interface detail showing PAE values for the 33 interface residues (21 from DLG1, 12 from ZDHHC5) identified by dual criterion of PAE < 4Å and spatial distance ≤ 8Å. Mean interface PAE is 5.3Å, with 45.3% of residue pairs showing PAE < 5Å, indicating a relatively confident prediction of the structured interface.

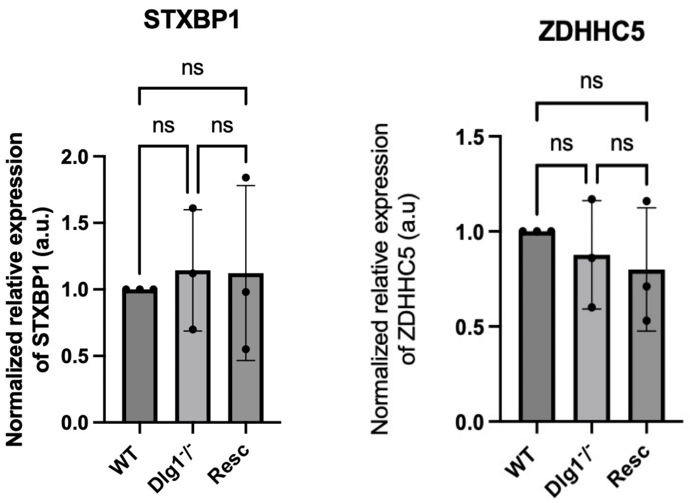

Figure S3. Relative cellular levels of STXBP1 and ZDHHC5 in IMCD3 cell lines. Related to Figure 3C. Quantification of relative cellular levels of STXBP1 and ZDHHC5 in the indicated cell lines, based on western blots (n=3). Graphs represent relative band intensities normalized to the WT. Statistical analysis was done using one-way ANOVA with Tukey's multiple comparison's test. ns, P-value>0.5.

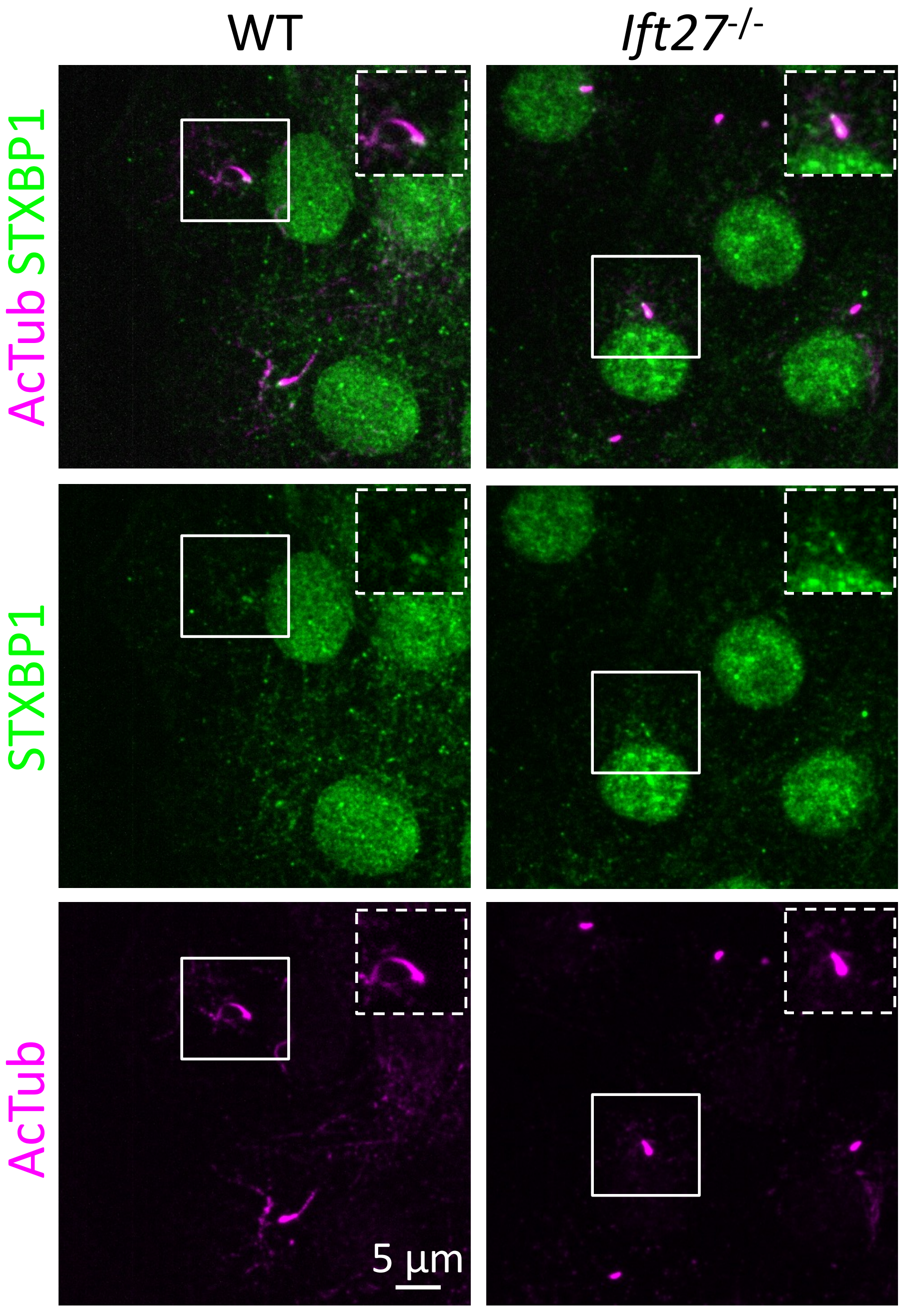

Figure S4. IFT27 regulates ciliary levels of STXBP1. Related to Figure 4. IFM analysis showing ciliary base localization STXBP1 in control (WT) IMCD3 cells, whereas *Ift27*$^{-/-}$ cells display accumulation of STXBP1 all along the cilium. Cells were serum-starved for 24 hours and subjected to IFM analysis using antibodies against STXBP1 (green) and acetylated α-tubulin (AcTub; magenta), which stains the ciliary axoneme. Insets show zoom-ins of the cilium-basal body axis.

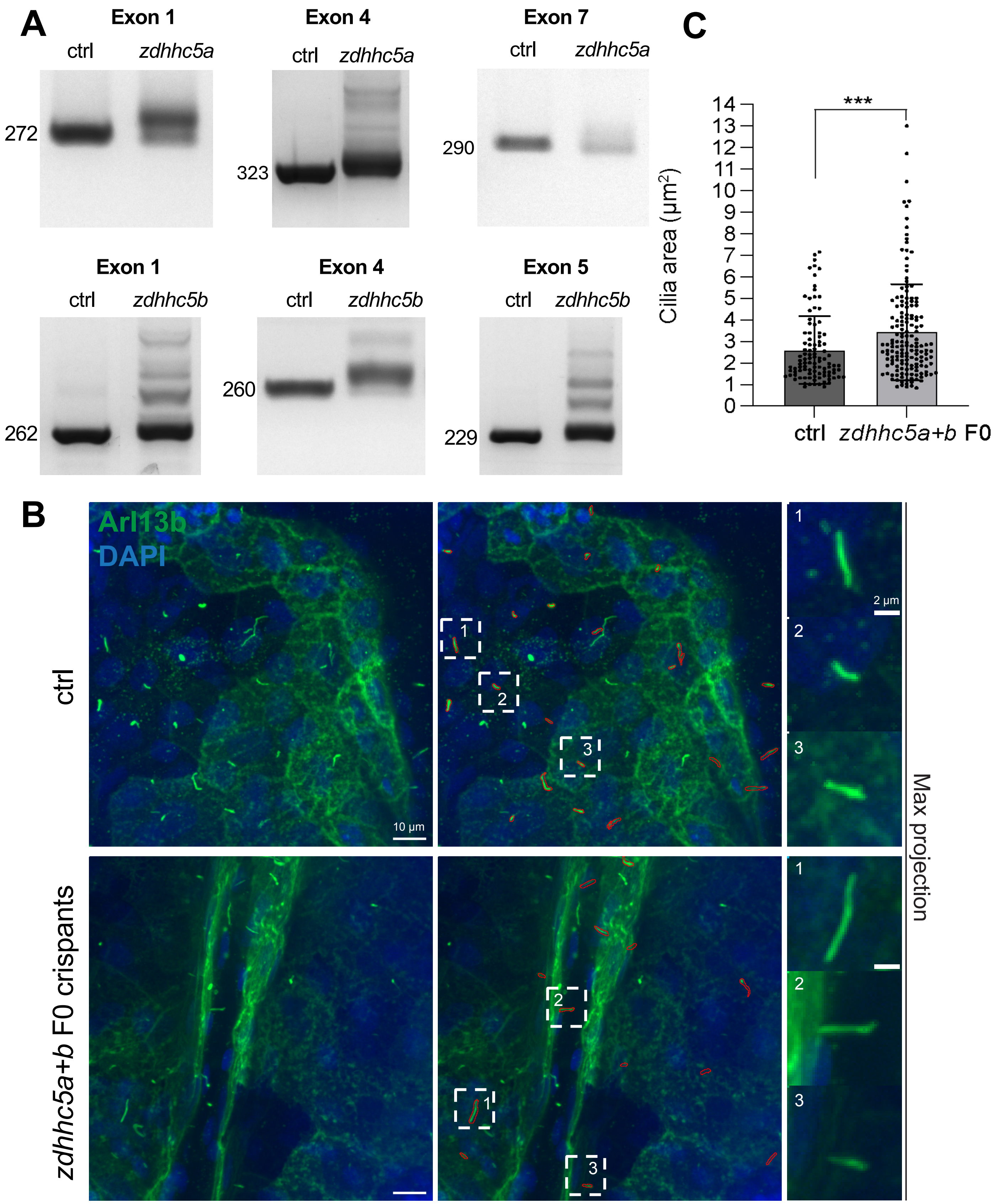

Figure S5. Depletion of *zdhhc5* isoforms in zebrafish causes ciliary elongation in the brain. Related to Figure 5 and Figure 6. (A) PCR analysis confirming efficient genome editing by the presence of heterogeneous indels in pooled zebrafish embryos injected with *zdhhc5*-targeting RNPs, relative to control RNP-injected embryos. **(B)** Confocal images of the brain region showing elongated primary cilia in *zdhhc5a+b* depleted F0 crispants compared to control RNPs injected *Tg(arl13b:GFP)* zebrafish (ctrl). **(C)** Comparison of the mean ciliary area between ctrl and crispant fish. ***, $p<0.001$ using non-parametric Mann Whitney test. Based on data from 3 ctrl fish (9 fields of view, 100 cilia) and 7 fish for *zdhhc5a+b* F0 crispants (12 fields of view, 158 cilia) in 1 experiment.
